# Impact of Training on Three-Dimensional versus Two-Dimensional Laparoscopic Systems on Acquisition of Laparoscopic Skills in Novices: A Prospective Comparative Pilot Study

**DOI:** 10.1155/2016/4197693

**Published:** 2016-11-22

**Authors:** Yasser A. Noureldin, Ana Stoica, Pepa Kaneva, Sero Andonian

**Affiliations:** ^1^Division of Urology, McGill University Health Center, McGill University, Montreal, QC, Canada; ^2^Department of Urology, Benha University Hospital, Benha University, Benha, Egypt

## Abstract

In this prospective educational study, 10 medical students (novices) were randomized to practice two basic laparoscopic tasks from the MISTELS program, namely, Pegboard Transfer (PT) and Intracorporeal Knot Tying (IKT) tasks, using either a 2D or a 3D laparoscopic platform. There was no significant difference between both groups in the baseline assessments (PT task: 130.8 ± 18.7 versus 151.5 ± 33.4; *p* = 0.35) (IKT task: 123.9 ± 41.0 versus 122.9 ± 44.9; *p* = 0.986). Following two training sessions, there was a significant increase in the scores of PT task for the 2D (130.8 ± 18.7 versus 222.6 ± 7.0; *p* = 0.0004) and the 3D groups (151.5 ± 33.4 versus 211.7 ± 16.2; *p* = 0.0001). Similarly, there was a significant increase in the scores of IKT task for the 2D (123.9 ± 41.0 versus 373.3 ± 47.2; *p* = 0.003) and the 3D groups (122.9 ± 44.9 versus 338.8 ± 28.6; *p* = 0.0005). However, there was no significant difference in the final assessment scores between 2D and 3D groups for both tasks (*p* > 0.05). Therefore, 3D laparoscopic systems do not provide an advantage over 2D systems for training novices in basic laparoscopic skills.

## 1. Introduction

Currently, laparoscopic interventions are considered the standard of care in different surgical subspecialties [1]. This might be attributable to better outcomes in terms of fewer blood loss, lower complications, quicker wound healing, and better cosmesis when compared with open surgery [2].

Laparoscopic surgery is traditionally performed using two-dimensional (2D) laparoscopes, which lack depth perception. Therefore, surgeons use indirect clues such as shading, motion parallax, and texture gradient to compensate for the lack of the third dimension [3, 4]. The da Vinci robotic system, introduced at the beginning of 21st century, offers three-dimensional (3D) visualization, intuitive motion, and additional degrees of freedom [5]. However, the high cost of these systems limits their availability to academic centers, with little access to novice trainees. Therefore, manufacturers were pressed to invent a less expensive 3D laparoscope that offered 3D visualization during conventional laparoscopic procedures and avoided the high cost of robotic systems.

Recent studies have shown that surgeons are exploiting these novel 3D laparoscopic systems as they offer better depth perception and spatial orientation [3–6]. However, these studies have not compared the effect of using 2D and 3D laparoscopic systems on performance during training. Therefore, the purpose of this study was to assess whether training using the 3D laparoscopic system results in improved performance compared with training using the 2D laparoscopic system among medical students (novices) without any previous laparoscopic experience. The hypothesis of this study was that novices who trained using the 3D laparoscopic system would perform better than novices who trained using the 2D laparoscopic system.

## 2. Materials and Methods

### 2.1. Study Population, Randomization, and Evaluations

**Figure 1:**
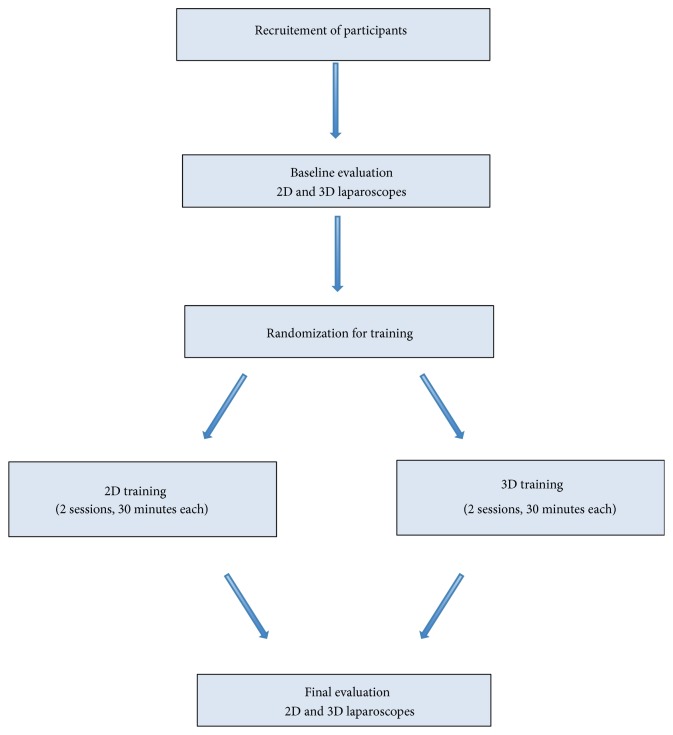
Study design.

After obtaining ethics approval from McGill University (IRB # A02-E05-15B), first- and second-year medical students (novices) were recruited to participate in this study from May 25th to June 5th, 2015. One of the authors (Ana Stoica) is a first-year medical student, who recruited her classmates to participate in this study voluntarily (without any compensation). Participants were interested to participate in this study to see first-hand what laparoscopic surgery was about. Therefore, all participants were novices, without any previous laparoscopic experience. All participants signed an informed consent prior to starting the study. By watching an instructional video, participants were introduced to the Pegboard Transfer (PT) and Intracorporeal Knot Tying (IKT) tasks of the McGill Inanimate System for Training and Evaluation of Laparoscopic Skills (MISTELS) program. During the PT task, each participant was asked to lift each triangular object off a peg from the left side of the pegboard using a Maryland grasper with the nondominant hand and transfer it to the Maryland grasper in the dominant hand and place it on another peg on the right side of the pegboard. There are a total of 6 objects and 12 pegs on the pegboard. Once all 6 objects are transferred to the right side of the pegboard, they are transferred back to the left side of the pegboard by grasping each with the dominant-hand Maryland grasper and transferring it to the nondominant-hand Maryland grasper. During the IKT task, each participant was asked to place a surgeon's knot through predetermined points on a Penrose drain [7]. The scoring of the PT task consists of the time required to complete the task (a cutoff time of 300 sec) with penalties for objects dropped outside of the field of view. Meanwhile the scoring of the IKT task consists of the time to complete the task (a cutoff time of 600 sec) with penalties for gaps and deviations from the predetermined points (both measured in mm) in addition to penalties for insecure knots [7]. They then proceeded to complete the two tasks using both 2D and 3D laparoscopic systems (baseline evaluation). Participants were then randomized for their training using either 2D or 3D laparoscopic system (Figure 1).

Participants were randomized into two groups (2D and 3D) using sealed envelopes. During training, participants watched again the instructional video and received feedback before, during, and after performing the task. Two training sessions were held where participants completed six times the PT task and four times the IKT task using either the 2D or the 3D depending on their randomized group. Feedback was standardized such that all participants received the same information. Following completion of training, final assessment of the two MISTELS tasks was performed for each participant using both 2D and 3D laparoscopic systems. All participants were evaluated during all sessions (baseline, training sessions, and final assessment) by a trained MISTELS evaluator and were scored according to what has been previously published [7–10]. Raw scores were used for data analysis. A questionnaire was administered after baseline and final assessments to determine preference of trainees regarding the 2D and 3D laparoscopic systems (qualitative assessment).

### 2.2. The 2D and 3D Laparoscopic Systems

A standard MISTELS training box was used for this study [11]. This system already comes with a High Definition (HD) 2D laparoscopic system [11]. For the 3D laparoscopic system, a 3D HD laparoscopic system was used (*Viking, La Jolla, CA, USA*). Participants used a passive, lightweight, polarized eyewear to perceive 3D images (micropolarization technology) (*Viking, La Jolla, CA, USA*). A 10 mm zero-degree laparoscope was used for both 2D and 3D laparoscopic systems.

### 2.3. Statistical Analysis

The IBM SPSS Statistics for Windows, Version 20 (Armonk, NY: IBM Corp.), was used for data analysis. Descriptive data were presented in terms of numbers and percentages or means and standard deviations. Comparison of continuous variables was performed using the Mann–Whitney *U*-test. Chi-Square test was used to compare categorical variables. The Wilcoxon Signed rank test was used to compare pretraining and posttraining scores. Two-tailed *p* values of <0.05 were considered statistically significant.

## 3. Results

A total of 10 medical students (novices) were recruited for this study with a mean age of 24.2 ± 2.70 years and female gender representing 50%. Eight participants were from first-year and two from second-year medical school. Participants were randomized into five trainees in each arm (2D versus 3D). The mean age was comparable between the 2D and 3D groups (24.8 ± 3.11 versus 23.6 ± 2.41 years; *p* = 0.52) (Table 1). Similarly, both groups were comparable in terms of female participants, right-handedness, and year of medical school (*p* values >0.05) (Table 1).

**Figure 2:**
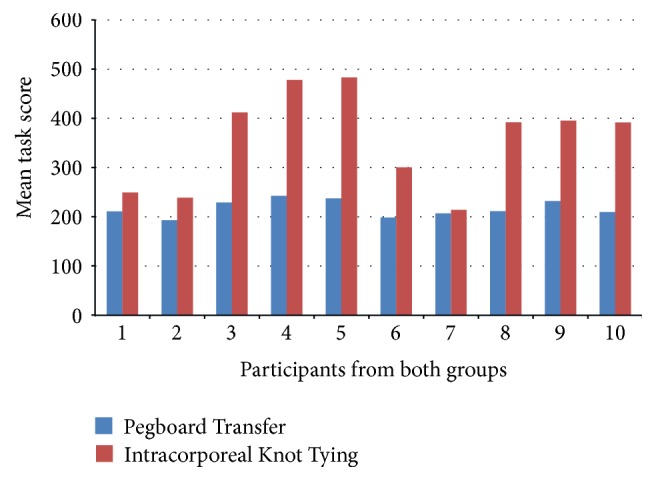
Mean scores of all participants for both tasks.

The mean scores of all participants from both groups are presented in Figure 2. There was no significant difference on the baseline assessments for both 2D and 3D groups in terms of their scores for PT task (130.8 ± 18.7 versus 151.5 ± 33.4; *p* = 0.350) and IKT task (123.9 ± 41.0 versus 122.9 ± 44.9; *p* = 0.986).

**Figure 3:**
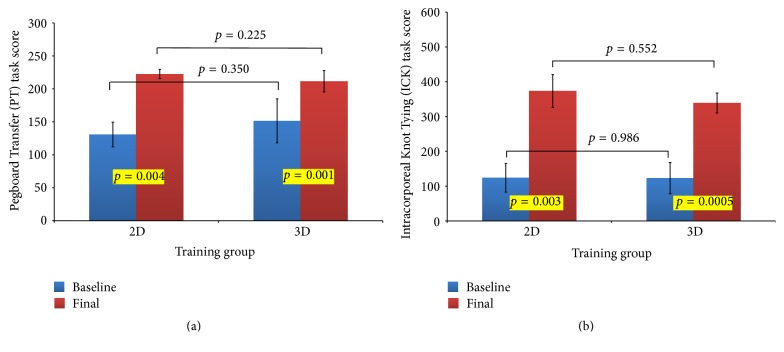
Comparison of mean scores for baseline and final evaluations. (a) Pegboard Transfer task and (b) Intracorporeal Knot Tying task.

After two training sessions, there was a significant increase in the scores of PT task for the 2D (130.8 ± 18.7 versus 222.6 ± 7.0; *p* = 0.0004) and the 3D groups (151.5 ± 33.4 versus 211.7 ± 16.2; *p* = 0.0001). Similarly, there was a significant increase in the scores of IKT task for the 2D (123.9 ± 41.0 versus 373.3 ± 47.2; *p* = 0.003) and the 3D groups (122.9 ± 44.9 versus 338.8 ± 28.6; *p* = 0.0005) (Figure 3). However, there was no significant difference in the final assessment scores between the 2D and 3D groups for both tasks (*p* values >0.05) (Figure 3).

**Figure 4:**
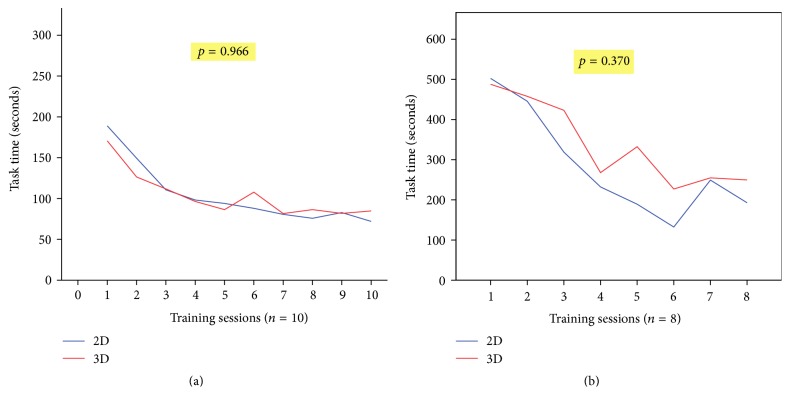
Time to complete each task during training sessions with 2D and 3D laparoscopic systems. (a) Pegboard Transfer task and (b) Intracorporeal Knot Tying task.

Since both groups improved with practice, there was no significant difference between the 2D and 3D groups in the mean PT task time over 10 consecutive assessments during the two training sessions (*p* > 0.05) (Figure 4(a)). Similarly, there was no significant difference between the 2D and 3D groups in the mean IKT task time over 8 consecutive assessments during the two training sessions (*p* > 0.05) (Figure 4(b)).

Initially, participants preferred the 3D system over the 2D system for the PT task while there was no clear preference for the IKT task (Table 2). Following the training sessions and final evaluations, participants preferred the 2D system for both PT and IKT tasks (Table 2).

## 4. Discussion

Over the last decade, there were several studies comparing 2D with 3D laparoscopic systems in terms of performance during laparoscopic training and surgery [1, 3, 12–20]. While some reported superiority of 3D laparoscopic systems [1, 3, 16–22], others showed that both systems were equally effective [11–14, 20]. In the current study, there were no significant differences neither in the learning curves nor in the final performance of novices using 2D and 3D laparoscopic systems for the PT and IKT tasks. This could be explained by the small sample size (5 participants in each arm) and the short period of training with subsequently low number of training sessions (two training sessions over two weeks). Another explanation could be the use of a HD 2D laparoscopic system, which has been reported to compensate for some of the deficiencies in 2D laparoscopic systems [23, 24]. In addition, participants complained that their movements were limited when using the heavy 3D laparoscopic system, which is almost double the size of the 2D laparoscopic system. These results are similar to a recent study by Mistry and colleagues [20]. They did not find a significant difference among medical students performing IKT task (*p* = 0.795). However, their findings regarding the PT task were different from the current study. They found significantly higher scores in favor of the 2D laparoscope (*p* = 0.001) [20]. The authors concluded that adding stereoscopic vision with 3D laparoscopic systems increased the cognitive load of novice medical students decreasing acquisition of new technical skills [20].

Other studies reported the superiority of 3D laparoscopic systems over 2D laparoscopic systems in terms of performance during PT and IKT tasks [1, 16]. This might be due to recruitment of more skilled participants (residents and expert surgeons) than the novice medical students recruited in the present study. Three other studies demonstrated superiority of the 3D laparoscopic system for precision in performing laparoscopic tasks [3, 25, 26]. However, the time to task completion was not significantly better with the 3D laparoscopic system [3, 25, 26]. This explains why there was no significant difference between the 2D and 3D groups in the present study since the MISTELS scoring system is mainly based on the time required to complete each task rather than precision, specially considering that the PT task was one of the two tasks tested.

Finally, a recent systematic review comparing 2D with 3D laparoscopic systems for laparoscopic surgery found contradictory results [6]. Studies that demonstrated the superiority of 3D laparoscopic systems over 2D laparoscopic systems [3, 18, 19] assessed the surgeons' comfort during laparoscopic procedures rather than assessing their performance on MISTELS. Therefore, it seems that using 3D laparoscopic systems is superior to 2D laparoscopic systems when expert laparoscopic surgeons perform laparoscopic procedures rather than when novice medical students are training on MISTELS.

Interestingly, in the present study, participants preferred the 3D favoring the 3D laparoscopic system at baseline evaluation while they opted for the 2D favoring the 3D laparoscopic system at the final evaluation (Table 2). Reasons given by participants for the preference of the 2D favoring the 3D laparoscopic system were eye straining in addition to dissimilarity to how they were used to viewing screens during daily living activities. Results of the present study suggest that 2D laparoscopic systems are preferred for novice trainees during the initial training and acquisition of basic hand-eye coordination skills. Furthermore, the significant improvements from baseline to final evaluation in novice trainees after only two training sessions point to the benefit of intensive training over a short period of time (two weeks). We think that the perspective of this study is that while 3D laparoscopic system is better for expert laparoscopic surgeons during real-life operations, it seems that it does not add much during the early phases of novice training. Therefore, this highlights the effectiveness of traditional training on widely available laparoscopic training boxes which use 2D laparoscopic systems and avoids the higher costs associated with the 3D laparoscopic systems for training novices.

Despite being a prospective study, several limitations are present such as the small sample size. Another limitation was the low number of training sessions (two sessions). However, both groups significantly improved their learning curves in both tasks, indicating comparable outcomes of the two systems for training of novices. Further studies are needed to recruit larger sample sizes with more training sessions.

## 5. Conclusion

Despite the small sample size and the low number of training sessions, this study showed a significant improvement in the performance of novices following training on MISTELS using both 2D and 3D laparoscopic systems. However, there was no significant difference in the final performance between the two laparoscopic systems.

## Figures and Tables

**Table 1:** Demographic information of participants.

Variable	Two-dimensional (*n* = 5)	Three-dimensional (*n* = 5)	*p* value
Age (years)	24.8 ± 3.11	23.6 ± 2.41	0.52
Female gender	1 (20%)	4 (80%)	0.21
First-year student	3 (60%)	5 (100%)	0.44
Right-handedness	5 (100%)	5 (100%)	0.99
Video game player	4 (80%)	1 (20%)	0.21
Interest in surgery	3 (60%)	2 (40%)	0.99
Observation of operative procedures	4 (80%)	2 (40%)	0.52
Use of corrective eye glasses/lenses	3 (60%)	5 (100%)	0.44

Data are presented in terms of mean ± SD or number (percentage), whenever appropriate.

**Table 2:** Confidence and preference of participants regarding 2D and 3D laparoscopic systems.

Statement	Evaluation		*p* value
	Baseline	Final	
How confident were you about your performance?			
Not at all	4 (40%)	0	0.267
Slightly unconfident	1 (10%)	2 (20%)	
Neutral	2 (20%)	3 (30%)	
A little	2 (20%)	4 (40%)	
Very	1 (10%)	1 (10%)	
Which laparoscopic system did you prefer to perform each task?			
Pegboard Transfer			
2D	3 (30%)	8 (80%)	**0.024**
3D	7 (70%)	2 (20%)	
Intracorporeal Knot Tying			
2D	5 (50%)	7 (70%)	0.208
3D	5 (50%)	3 (30%)	

## Abbreviations

2D: Two-dimensional
3D: Three-dimensional
BLUS: Basic laparoscopic urologic skills
FLS: Fundamentals of laparoscopic surgery
HD: High definition
IKT: Intracorporeal Knot Tying
MISTELS: McGill Inanimate System for Training and Evaluation of Laparoscopic Skills
PT: Pegboard Transfer.
